# RNAi mediated myosuppressin deficiency affects muscle development and survival in the salmon louse (*Lepeophtheirus salmonis*)

**DOI:** 10.1038/s41598-019-43515-w

**Published:** 2019-05-06

**Authors:** Anna Z. Komisarczuk, Heidi Kongshaug, Ming Li, Frank Nilsen

**Affiliations:** 1 Sea Lice Research Centre, Department of Biological Sciences, University of Bergen, Thormøhlensgate 53 A/B, 5008 Bergen, Norway; 2 Key Laboratory of Aquaculture Disease Control, Ministry of Agriculture, and State Key Laboratory of Freshwater Ecology and Biotechnology, Institute of Hydrobiology, Chinese Academy of Sciences, Wuhan, 430072 China

**Keywords:** Disease model, RNAi, Animal physiology

## Abstract

Muscle activity is regulated by stimulatory and inhibitory neuropeptides allowing for contraction and relaxation. In Arthropods, one of the important myoinhibitors is Myosuppressin, belonging to FMRFamide-like peptides, that was shown to have inhibitory effects on visceral muscle contraction and to regulate vital physiological processes including reproduction or feeding. We have identified myosuppressin in salmon louse *Lepeophtheirus salmonis* (*Lsal*MS) and systematically characterised its function and complex abnormalities emerging after *Lsal*MS knockdown by RNAi in all developmental stages in this species. Immunohistochemistry analysis localized the *Lsal*MS mainly to the central nervous system, but also to the vital organs within the alimentary tract and the reproductive system. The most striking feature of *Lsal*MS deficiency during lice development was severe reduction of the muscle content, with abnormalities detected in both the visceral and skeletal muscles. Moreover, down-regulation of *LsalMS* affects moulting, spermatophore deposition and feeding by affecting development of the intestinal wall and increasing its contraction frequency.

## Introduction

The salmon louse, *Lepeophtheirus salmonis*, (Copepoda: Crustacea), is a marine ecto-parasite of Atlantic salmon (*Salmo salar*) that causes large economic loss to the farmed *salmonids*^1^ and constitute a major threat to the wild fish populations^2^. Salmon louse feeds on the host’s mucus, skin and blood, decreasing fish growth and causing lesions that constitute the gateway for other pathogens^3^. Until now, fight against salmon louse has been based mainly on the medicinal treatment and various physical methods^4^. However, the medicine-based delousing strategy has progressively lost the efficacy, since the lice have developed resistance against the various chemotherapeutics^5^, while, physical methods cause stress and damage to the fish, leading to high post-treatment mortality. Therefore, there is an urgent need to develop new and efficient means to control salmon louse infections.

In recent years, increased interest has been aimed at endocrine-based pest control strategy that involves development of drugs targeting neuropeptides in invertebrates^6–8^. Neuropeptides, their receptors and enzymes involved in their metabolism, have long been considered as potential targets for the development of new chemotherapeutics, due to their various physiological and behavioural functions, conserved structure and widespread distribution in Arthropods, and the possibility that such targets could deliver specific and environment friendly treatment^9–13^. However, until now, no commercial insecticide utilising neuropeptide’s agonists or antagonists has been discovered or used, mainly due to the missing information and lack of full understanding of neuropeptide and their receptors function and structure. To strengthen the development of such pest control strategy, knowledge about neuropeptide composition, their biological activities and receptor-neuropeptide interactions is of critical importance^9–13^.

Neuropeptides are an important class of messengers and hormones acting as critical signal transducers and modulators in a wide range of developmental, physiological and behavioural processes. The variety of functions neuropeptides play in the animals suggest that alternations in peptide expression, synthesis, or signalling could result in abnormalities or impaired function resulting in decreased fitness or death of parasite such as the salmon louse.

One of the important neuropeptides found in Arthropods’ genomes is Myosuppressin (MS). Numerous myosuppressin precursors have been identified through cloned DNA or predicted from the genomes in a wide range of invertebrates^14–19^. The first myosuppressin was isolated from the extracts of the nervous tissues of the cockroach *Leucophaea maderae* (leucomyosuppressin, LMS – pQDVDHVFLRFamide)^20,21^, and subsequently identical decapeptides were isolated from other cockroaches^22,23^. Similar decapeptides, which differ from LMS by only one amino acid residue in the N-terminal end of the peptide, have been isolated and sequenced in the flies *Neobellieria bullata*^24^ and *Drosophila melanogaster* (dromyosuppressin, DMS)^25^, the beetle *Zophobas atratus*^14^, and the locusts *Schistocerca gregaria* and *Locusta migratoria*^26–31^.

Myosuppressin belongs to the FMRFamide-like peptides family with the consensus decapeptide sequence at the C-terminal end of X_1_DVX_4_HX_6_FLRFamide (where X_1_ = pQ, P, T or A, X_4_ = D, G or V, and X_6_ = V or S)^32–34^. The last six amino acids of the decapeptide constitute the active core that is identical in all Arthropods studied so far and sufficient for antimyotropic activity^30^. Myosuppressin decapeptides mediate their function by binding to myosuppressin receptors that belong to the G-protein coupled receptors^35,36^.

Myosuppressin belongs to the myoinhibitory peptides and was shown to have pleiotropic functions in regulating activity of the visceral muscles^14,21,25,37–39^. Apparently, it plays an important role in digestion by inhibiting peristaltic contraction of the alimentary tract^27,40–42^, and food intake^23^. It stimulates invertase and α-amylase release in the midgut^22,43,44^, and increases free sugar level in the haemolymph^45^. It plays an important role in reproduction by inhibiting muscles responsible for ovulation and egg-laying^14,20,24,27,28,30,31,41,42,46–49^, and the contractions of the ejaculatory duct^14^. Furthermore, myosuppressin decreases cardiac contractility^20,31,39^ in a dose-dependent manner^37,39,40,50^. Recently, it was shown that myosuppressin from *Mamestra brassicae* is involved in regulation of prothoracic glands activity and plays an important role in the initiation and maintenance of pupal diapause^51^.

The majority of publications describe structure-activity relationship and function of myosuppressin in controlling various muscles, whereas, myosuppressin’s role in development, and effects of its deficiency or dysfunction are poorly investigated. Thus, we investigated myosuppressin function in the animal development in a number of developmental stages of salmon louse *Lepeophtheirus salmonis* (*Lsal*MS), from free-living planktonic stages to parasitic stages. We knocked-down *Lsal*MS by RNA interference at various developmental time points and looked for developmental and behavioural abnormalities. Using MS-specific antisera and quantitative qRT-PCR we mapped gene expression and peptide localization to nervous system, reproductive system and alimentary tract.

## Results

### Sequence analysis and molecular phylogeny of the *L. salmonis* myosuppressin

**Figure 1:**
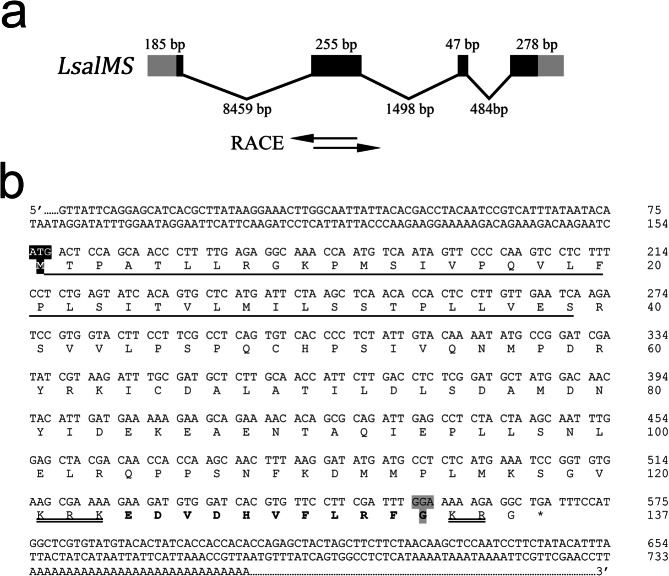
Genomic organization and identified myosuppressin encoding cDNA sequence from *Lepeophtheirus salmonis*. (**a**) Schematic representation of the genomic organization of the *Lepeophtheirus salmonis* myosuppressin gene *Lsal*MS. GenBank ID: MH748227. The numbers refer to the size of the exons and introns in base pairs. Coding sequence of *LsalMS* gene consists of 4 exons. The open reading frame (ORF) is shown as black boxes and 5′ and 3′ UTR sequences as grey. Introns are shown as lines between exons. Below: primers used to generate PCR products by RACE with 279 bp overlapping sequence indicated by black arrows. The overlapping fragment was used as a template for dsRNA used in RNAi. (**b**) Nucleotide cDNA sequence and deduced amino acid prepropeptide of the *LsalMS* gene. The coding region starts at the nucleotide sequence ATG (black) and stops at the sequence TAA (denoted by an asterisk). The signal peptide is underlined and atypical putative proteolytic processing sites that flank decapeptide are double underline. The mature *Lsal*MS is depicted in bold and the C-terminal glycine residue of decapeptide required for amidation is highlighted in grey.

**Figure 2:**
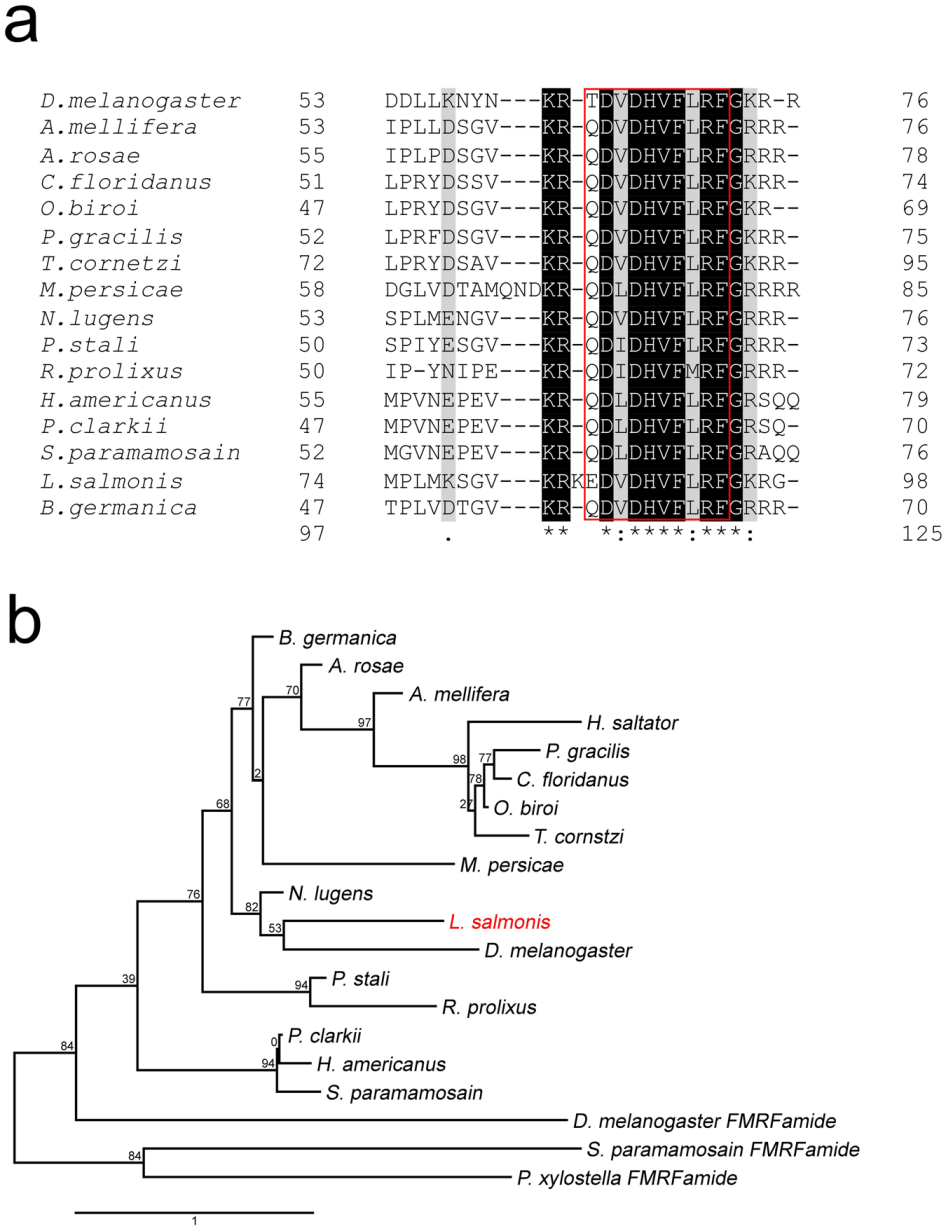
MS propeptides multiple sequence alignment and phylogenetic analysis. **(a**) C–terminal part of the protein alignment of myosuppressin propeptide sequences from different Arthropods species. Propeptide sequences (accession numbers Table 1) were used to generate multiple sequence alignment with ClustalOmega. Identical amino acids are highlighted in black and similar amino acids are highlighted in grey. Databases corresponding to the mature peptide are in a red frame (**b**) Analysis of phylogenetic relationship of *Lepeophtheirus salmonis* myosuppressin propeptide with sequences from other Arthropods generated by Bayesian method. *Lsal*MS in red. FMRFamide sequences of Arthropods species were used as an outgroup. Branch numbers represent bootstrap values in percent and bar represents 0.2 substitutions per site. Branch lengths are proportional to sequences divergence. The accession numbers listed in Table 1.

The gene encoding myosuppressin in *Lepeophtheirus salmonis* (*LsalMS*) was identified by the standard Blastp search of the collection of protein predictions from expressed sequence tags (EST) (sealouse.imr.no) using *Drosophila melanogaster* myosuppressin peptide (AAF56283.1) as the query sequences. The result revealed one candidate with low e-value (e = 7e-08). To obtain the full cDNA sequences of the identified gene, we carried out 5′ and 3′ Rapid Amplification of cDNA Ends (RACE) using PCR primers specific for exon sequences obtained from sealouse.imr.no database (Supp. Table 1). Genomic organisation of *LsalMS* is shown on Fig. 1a. The final *Lsal*MS sequence has been submitted to GenBank (GenBank accession number: MH748227). The full cDNA sequence of *LsalMS* is 763 nucleotides long. It contains a 5′-UTR of 154 bp and a 3′-UTR of 195 bp, including a polyA. The deduced open reading frame is 414 nucleotides long and encoded a prepropeptide of 137 amino acid residues. A signal peptide was identified with the cleavage site located between the 39 and 40 amino acid (S/R), giving a precursor peptide of 98 aa. The deduced amino acid sequence of the decapeptide, identified based on multiple sequence alignment, is flanked by abnormal proteolytic processing sites, tribasic (KRK) and dibasic (KR) sites located before and after the decapeptide^52^ (Fig. 1b). Thus, the encoded *L. salmonis* decapeptide begins with glutamic acid (E) and ends with a phenylalanine residue (F), and it follows the consensus pattern for myosuppressin peptides (Figs 1b and 2a). In the majority of insects^14,20,22–26,29^, the mature decapeptide starts with glutamic acid (E) or glutamine (Q), and both amino acids can be changed to pyroglutamic acid during post-translational modifications^53,54^. The glycine residue at the end of the decapeptide is necessary for amidation by peptidylglycine α-amidating monooxygenase^55^. The final sequence of *L. salmonis* mature peptide is predicted to be pEDVDHVFLRFamide. No transmembrane regions, specific functional domain, or N-glycosylation sites were detected in the predicted peptide sequence.

The alignment of myosuppressin propeptide with propeptides from other species revealed the same organization, with a similar length of 70–100 amino acids. The alignment also showed that the greatest identity is concentrated in the C-terminal region, which corresponds to the mature decapeptide (Fig. 2a). Comparison of full prepropeptide *LsalMS* cDNA sequence with the genomic sequence of *L. salmonis* showed the presence of four exons in the *LsalMS* gene (Fig. 1a).

The *Lsal*MS peptide precursor was compared with those of other species, revealing 38% identity to *Drosophila melanogaster*, 55% to the Hemiptera *Nilaparvata lugens*, and 34%, 35% and 37% to other Crustaceans, *Scylla paramamosain*, *Homarus americanus* and *Procambarus clarkia* respectively. The *Lsal*MS decapeptide has identical sequence to Zopat-MS and differs from LMS (leucomyosuppressin, the first identified myosuppressin from the cockroach *Leucophaea maderae*) by only one amino acid residue at the N-terminal end.

The propeptide sequences were used to construct a phylogenetic tree (for accession numbers see Table 1), shown in Fig. 2b. FMRFamide peptides were used as an out-group. In the rooted bootstraped tree, *Lsal*MS groups together with insects, the sister group to the Crustaceans.

**Table 1:** Amino acid sequences of Arthropods propeptides used in this study.

Subphylum	Class	Order	Species	Common name	Accession number
**Myosuppressin**					
Hexapoda	Insecta	*Diptera*	*Drosophila melanogaster*	Fruit fly	AAF56283.1
		*Lepidoptera*	*Tribolium castaneum*	Red flour beetle	EFA12055.1
		*Hymenoptera*	*Apis mellifera*	European honey bee	ACI90290.1
			*Athalia rosae*	Turnip sawfly	XP_012269698.1
			*Camponotus floridanus*	Florida carpenter ant	EFN71919.1
			*Dufourea novaeangliae*	Bee	KZC14232.1
			*Harpegnathos saltator*	Indian jumping ant	EFN78200.1
			*Melipona quadrifasciata*	Stingless bees	KOX74891.1
			*Ooceraea biroi*	Clonal raider ant	EZA55950.1
			*Pseudomyrmex gracilis*	Elongate twig ant	XP_020286321.1
			*Trachymyrmex cornetzi*	Fungus-growing ant	KYN27743.1
			*Trachymyrmex septentrionalis*	Fungus-growing ant	KYN37707.1
		*Hemiptera*	*Myzus persicae*	Green peach aphid	XP_022172677.1
			*Nilaparvata lugens*	Brown planthopper	BAO00963.1
			*Plautia stali*	Stink bug	BAV78815.1
			*Rhodnius prolixus*	Assassin bugs	ACT98134.1
		*Blattodea*	*Blattella germanica*	German cockroach	CAF04070.1
	Entognatha	*Collembola*	*Orchesella cincta*	Springtails	ODN03833.1
Crustacea	Malacostraca	*Decapoda*	*Homarus americanus*	American lobster	ACX46385.1
			*Procambarus clarkia*	Red swamp crawfish	BAG68789.1
			*Scylla paramamosain*	Mug crab	ALQ28580.1
	Maxillopoda	*Siphonostomatoida*	*Lepeophtheirus salmonis*	Salmon louse	MH748227
**FMRFamide**					
Hexapoda	Insecta	*Diptera*	*Drosophila melanogaster*	Fruit fly	NP_523669.2
		*Lepidoptera*	*Plutella xylostella*	Diamondback moth	AJM76779.1
Crustacea	Malacostraca	*Decapoda*	*Scylla paramamosain*	Mug crab	ALQ28593.1

Sequences were downloaded from NCBI.

### Ontogeny of *LsalMS*

**Figure 3:**
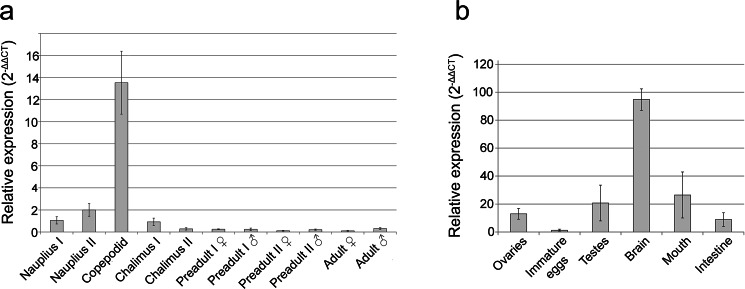
Relative expression of *LsalMS* in all *L. salmonis* developmental stages and tissues. (**a**) Calculated with 2^−ΔΔCT^ formula. The highest expression is detected in planktonic copepodid stage. Parasitic stages show lower expression of *LsalMS* than the free-living stages. From chalimus I stage, transcription level decreases with progression of the life cycle. N = 4 for each stage. The relative expression of *L. salmonis* myosuppressin genes in nauplius I was set to 1. Columns demonstrate relative expression and error bars present the standard deviation. (**b**) Relative expression of *LsalMS* transcript in various adult tissues was calculated with 2^−ΔΔCT^ formula. The highest level of transcript was found in the brain. *LsalMS* expression can be also detected in other tissues, which proper function is regulated by Myosuppressin: intestine and reproductive system. N = 4 for each tissue type. The relative expression of *LsalMS* in immature eggs was set to 1. Columns demonstrate relative expression level and error bars show the standard deviation.

Quantitative real-time PCR was used to analyse the ontogenic expression of *LsalMS* in all *L. salmonis* stages. Salmon louse has a complex development cycle consisted of eight stages: three free-living larval stages (nauplius I, II and copepodid) and five parasitic stages, attached to the fish (chalimus I, II, preadult I, II and adult)^56^. The level of *LsalMS* in nauplius I was set to 1 and considered as a baseline for quantification of expression in other stages. The lowest expression level of *LsalMS* transcripts was detected in adult females, whereas the copepodids showed the highest expression levels (Fig. 3a). In chalimus I, the first tested parasitic stage, the level of transcript decreased dramatically and it reached only 7% of the expression observed in the planktonic copepodids. In chalimus II, the transcript level decreased even more (50% of the value observed in chalimus I) and it persisted on this level in all following parasitic stages (Fig. 3a).

### qRT-PCR of *LsalMS* on tissue extracts from adult lice

Quantitative RT-PCR showed the highest *LsalMS* expression in the brain. Lower expression was detected in the mouth, intestine and parts of the reproductive system, mainly in the ovaries and testes and in female genital segment. Very low expression was detected in immature eggs extracted from female genital segment (set as 1 and used as a baseline for the whole analysis), which indicates that *LsalMS* transcripts detected in the genital segment were localised mainly in the oviduct (Fig. 3b).

### *LsalMS* peptide detection

**Figure 4:**
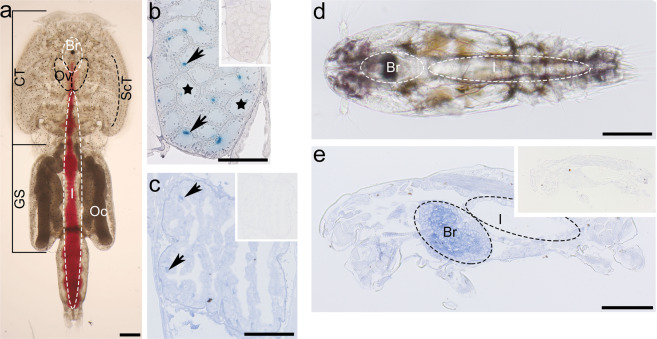
Tissue specific localization of *Lsal*MS. (**a**–**c**) Localization of *Lsal*MS neuropeptide in adult females. Insets present negative controls. (**a**) Dorsal view of adult female. The brain and ovaries are located in the cephalothorax (CT). Just under the brain, on the ventral side of the animal, the mouth is located. The intestine filled with fresh fish blood is visible along the whole animal, from the mouth in the cephalothorax, through the genital segment to the distal intestine opening. Subcuticular tissue is located along the body flanks in the cephalothorax. (**b**,**c**) Coronal section of the adult female. Positive immunostaining, indicated by arrows, is visible in small areas in the ovary (**c**) and intestinal wall (**b**). The light of tubules in the ovary is marked by asterisk (**b**). (**d**) Dorsal view of a copepodid. The brain area and the intestine are indicated. (**e**) The sagittal section of copepodid. Peptide is detected in the inner zone of the brain. Abbreviations: cephalothorax (CT), genital segment (GS), subcuticular tissue (ScT), brain (Br), intestine (I), ovaries (O), oocytes (Oc). Scale bar = 1 mm (**a**), 50 μm (**b**,**c**), 100 μm (**d**,**e**).

Immunohistochemistry analysis was performed to identify the localization of *Lsal*MS peptide in copepodids, adult female and male lice. In all tested stages, the specific signal was detected in the brain, in the internal area consisting of axons (Fig. 4c). In the adults, *Lsal*MS peptide was also detected in the intestine and reproductive organs, in cells that might have neural or endocrine origin (Fig. 4b,c). However, the exact cell type needs to be determined further.

### RNAi of *LsalMS* in copepodids

**Figure 5:**
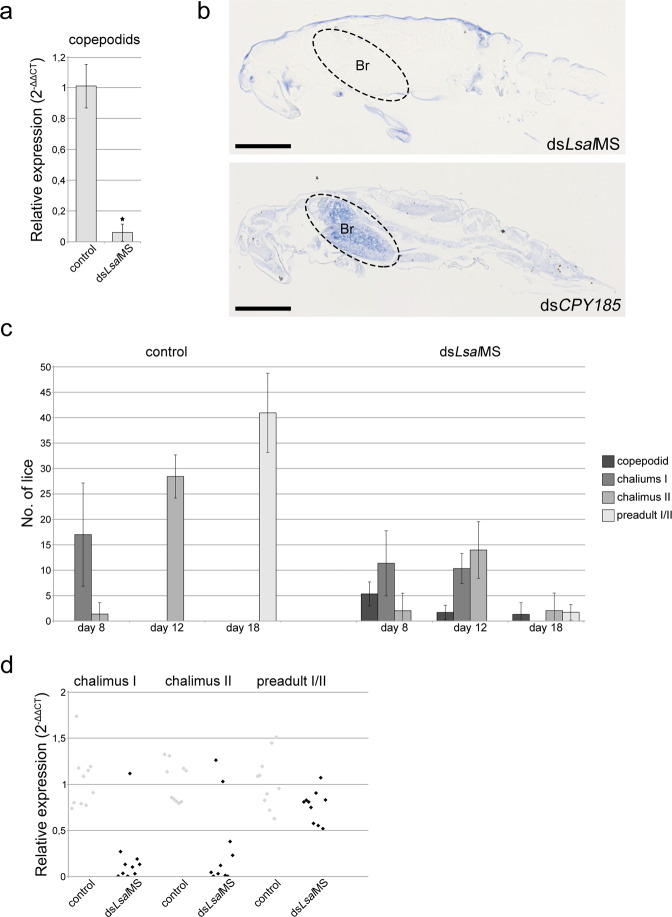
RNAi experiment on larvae. (**a**) Relative expression of *LsalMS* in copepodids after soaking with dsRNA at nauplii I stage, calculated with 2^−ΔΔCT^ formula. The relative expression of *LsalMS* in controls was set to 1. Columns demonstrate mean expression level and error bars indicate the standard deviation. Asterisks point to significant difference (p = 9.3e^−13^, n = 4 for each treatment group). (**b**) Localization of *Lsal*MS neuropeptide in a copepodids after RNAi experiment. Sagittal section. Peptide is detected in the brain (circled), in the control animals while no MS positive cells are present in ds*LsalMS* treated animals. Blue staining around the animal present on both images is the unspecific stain of cuticula. Scale bar = 100 μm. (**c**) Numbers and developmental stages of lice collected at different time points. Animals were collected from fish at 8, 12 and 18 day post infection (at 12 °C) (n = 3 fish for each time point and treatment). Numbers of the collected animals at day 8 and 12 post infection were similar in control and ds*LsalMS* treated group (day 8: control 18,3 (±8) vs. ds*LsalMS* 17,7 (±5,5), day 12: control 28,3 (±4,2) vs. ds*LsalMS* 26 (±6)), while number of lice collected on day 18 is much lower in ds*LsalMS* treated group than in controls (in average 41 (±7,9) controls vs. 5 (±1,5) ds*LsalMS* treated, p value = 0.001, n = 3 for each group). The developmental delay could be observed in case of ds*LsalMS* treated animals at all time points, with copepodids presented on fish even after 18 days post infection. (**d**) Relative expression of *LsalMS* in single chalimus I/II/preadult I lice collected from fish calculated with 2^−ΔΔCT^ formula. Lice larvae were soaked in dsRNA prior to infection of fish. Single chalimus I and II with normal *LsalMS* level were identified in ds*LsalMS* treated group, while all collected preadults from ds*LsalMS* treated group had *LsalMS* expression on similar level as in the control group. Each dot shows expression level of a single animal. The relative expression of *LsalMS* in controls was set to 1.

RNA interference was used to assess effects of *LsalMS* down-regulation in the larval stages. dsRNA was introduced by soaking during moult from nauplius I to nauplius II. After moulting, animals were left to develop to the copepodid stage and then investigated for any phenotypic defects. Although, the qRT-PCR analysis revealed significant down-regulation (more than 90%) of *LsalMS* in larvae treated with ds*LsalMS* in comparison to the control animals (Fig. 5a), no visible morphological or behavioural abnormalities were observed. However, immunohistochemistry with *Lsal*MS antisera showed substantial reduction in staining intensity in the ds*LsalMS* treated copepodids’ brain, indicating decreased level of the peptide (Fig. 5b).

While animals treated with ds*LsalMS* showed no visible phenotype at the free-living copepodid stage, we have investigated if down-regulation of *LsalMS* could have any effect on attachment ability and further development of the lice. Copepodids from each treatment group were used to infect fish (n = 3 for each treatment group). Development of lice was followed daily by visual observation. While, the control animals developed normally, ds*LsalMS* treated animals were able to reach chalimus II stage, but none of them were able to reach preadult I stage. The experiment was terminated after 30 days when all ds*LsalMS* treated lice had disappeared from the fish.

The infection experiment was repeated in order to investigate phenotypic defects in chalimus stages caused by ds*LsalMS* treatment. In total, nine fish were infected with lice from each treatment group. Lice were collected after 8, 12 and 18 days post infection (n = 3 fish per time point, per treatment group), when control lice were in the middle of chalimus I, chalimus II and preadult I/II stage, respectively. Animals were counted, photographed and carefully examined for potential morphological abnormalities. Similar infection rates were observed in both treatment groups, where in average 18 animals were recovered from each fish at day 8 (control 18,3 (±8) vs. ds*LsalMS* 17,7 (±5,5)) and 27 animals at day 12 post infection (control 28,3 (±4,2) vs. ds*LsalMS* 26 (±6)) (Fig. 5c). While control animals developed normally, delayed development was observed in ds*LsalMS* treated group, and copepodids were still present at day 12 post infection. A significant difference (p value = 0.001, n = 3 for each group) in the number of collected lice was obvious at day 18 post infection, where 96% less ds*LsalMS* treated lice were recovered from fish than controls, and only 34% of these were at the preadult stage (Fig. 5c).

Down-regulation efficiency was evaluated in lice collected at each time point by qRT-PCR. Comparison of *LsalMS* transcript level in ds*LsalMS* treated lice was done with controls in the corresponding developmental stage. Since copepodids were not present in the control group, they were excluded from analysis. To obtain sufficient number of preadult animals, infection was repeated (100 copepodids per fish, 6 fish used), and all lice were collected on day 18 post infection. On day 8, transcript level was tested in chalimus I (n = 10 for each group), on day 12 in chalimus II (n = 10 for each group) and on day 18 in preadult I males and females (n = 5♀/5♂ for each group). While majority of ds*LsalMS* treated chalimus I and II revealed significant down-regulation of transcript level, few animals (20% chalimus I, 10% chalimus II) showed *LsalMS* transcript levels comparable to controls. On the other hand, all recovered preadult I lice from ds*LsalMS* treatment group showed expression level similar to control group (Fig. 5d), suggesting that only animals with insufficient *LsalMS* down-regulation were able to survive and progress with development. Thus, the knock-down of *LsalMS* clearly affects normal developmental progress of the lice.

### RNAi of *LsalMS* in adults

**Figure 6:**
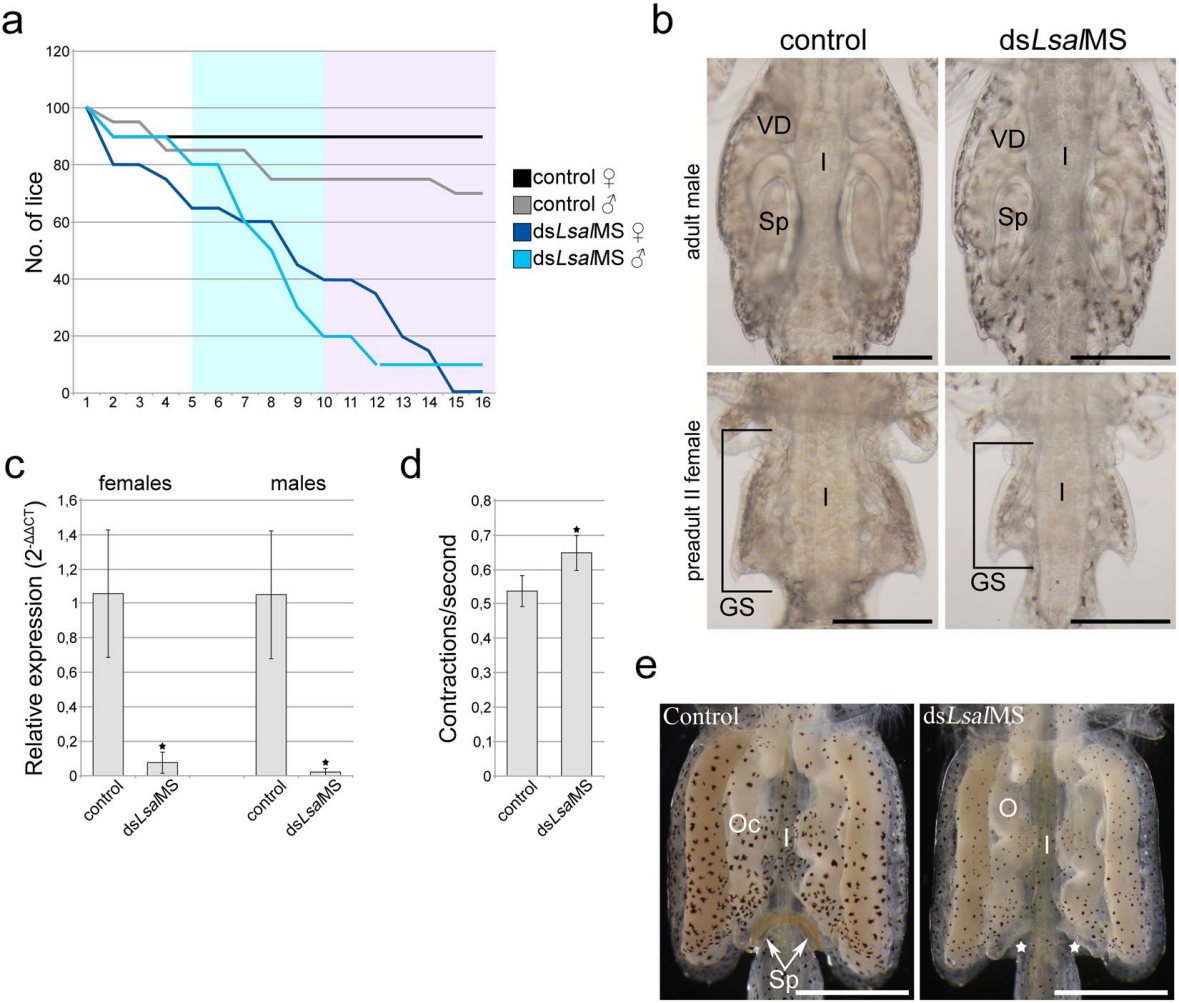
RNAi experiment on preadult lice. (**a**) Percentage of lice present on the fish during RNAi experiments. dsRNA was injected to preadult I males and females (n = 160 for each sex). 90% of control females and 70% of control males were still present on the fish at the end of experiment. Only 10% of ds*LsalMS* injected males and none of the females were on the fish at the end of experiment. None of the ds*LsalMS* injected females reached adulthood. (**b**) Genital segments of adult males and preadult II females, control and ds*LsalMS* injected. In males, vasa deferentia (VD) seem swollen and the spermatophores (Sp) are much smaller than in control lice. The preadult II female genital segment (SG and marked with black frame) of ds*LsalMS* injected lice was much smaller than the controls. In all animals, the intestine (I) is visible in the center. Scale bar = 100 μm. (**c**) Relative expression of *LsalMS* in preadult II females/adult males collected from fish/filters after dsRNA injection calculated with 2^−ΔΔCT^ formula. Columns demonstrate mean expression level and error bars indicate the standard deviation. Asterisks indicate significant difference (females: p = 0.0004, n = 5, males: p = 0.0002, n = 5). The relative expression of *LsalMS* in controls was set to 1. (**d**) Average rate of intestinal contractions in the preadult II females treated with dsRNA. Significantly higher contraction rate could be observed in females injected with ds*LsalMS* than in control lice: 0.64 contractions/s ds*LsalMS* lice vs. 0.53 contractions/s in control animals (n = 12 for ds*LsalMS* and n = 10 for controls). Columns show mean contraction rate per second and error bars demonstrate the standard deviation. Asterisks indicate significant difference (p = 0.0005). (**e**) Genital segments of untreated, adult females after mating with control or dsRNA treated males. Genital segments are full of oocytes (O), the intestine (I) is visible in the center. Spermatophores (Sp) are clearly visible on the female mated with control males while no spermatophores are deposited on female mated with ds*LsalMS* treated males. Typical spermatophore attachment places indicated with white asterisks. Scale bar = 200 μm.

To investigate phenotypic defect in adult animals, we injected preadult I animals of both sexes with dsRNA. In total, 160 pre-adult I female and 160 pre-adult I male lice were injected with ds*LsalMS*, in addition to animals injected with control dsRNA (four separate experiments, in total eight fish for each fragment). Animals were placed on the fish and observed daily. Lost animals were collected from the filters at the water outlet. The experiment was ended after 16 days, when all ds*LsalMS* treated females were lost from the fish and only a few males were still visible. In average, 70% of control females and 90% of control males were recovered from the fish, while only 10% of ds*LsalMS* treated males were still on the fish at the termination. The majority of female lice were lost at the preadult II stage or during moulting to adult stage (Fig. 6a). None of them were able to successfully complete moult to the adult stage.

While the control animals developed normally, they were able to swim and successfully attach to the fish and flat surfaces, ds*LsalMS* treated animals failed to do so. In all ds*LsalMS* treated animals (collected from filters and fish) the intestine wall appeared thinner, with numerous granules and gut content was reduced or absent. In adult males, spermatophores were smaller and vasa deferentia appeared swollen (Fig. 6b). The *LsalMS* transcript level in lice collected from fish/filters, evaluated by qRT-PCR, was 90–95% lower than in control animals (Fig. 6c). Close examination of the intestinal movement of recovered ds*LsalMS* treated preadult II females revealed significantly increased frequency of contractions (p = 0.0005) (Samples of the movies in Supp. Video 1). While in control lice 0.53 contraction per second was observed (n = 10), 0.64 contractions per second were counted in lice injected with ds*LsalMS* (n = 12) (Fig. 6d).

To evaluate mating potential of adult males, pre-adult I male lice were injected with ds*LsalMS* or control dsRNA, and placed together with non-injected females. The experiment was terminated after 6 weeks when all males reached adult stages and all control females had developed egg strings. In total, four males (13.3%) were recovered from the ds*LsalMS* group in comparison to 13 (43.3%) in the control group. Whereas all control females were mated, had spermatophores attached to the genital segment, and extruded normal, fertilized egg strings, with viable offspring, none of the females placed together with ds*LsalMS* injected males had spermatophore attached (Fig. 6e), and all collected egg strings (3 pairs) were not fertilized. However, typical mating behaviour, evident as pairing with females was observed in males treated with ds*LsalMS*.

**Figure 7:**
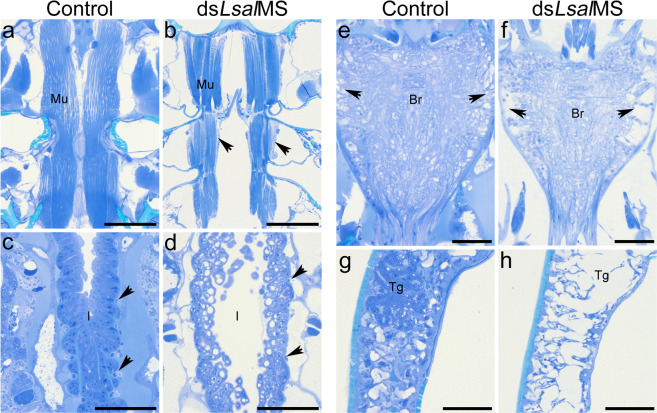
Histological analysis of adult males and preadult females after RNAi treatment. For description of anatomical structures in adult female lice see Fig. 4a and in adult male lice see Fig. 8a. Sections of control animals (**a**,**c**,**e**,**g**) and ds*LsalMS* treated animals (**b**,**d**,**f**,**h**). Coronal sections. Images are taken in the corresponding locations in the control and the ds*LsalMS* treated animals. (**a**,**d**) Adult males, (**e**,**h**) preadult II females. (**a**) Properly formed longitudinal muscles that connect the cephalothorax and genital segment in control animals, and (**b**) reduced muscle tissue. Arrows indicate ectopic tissue present in the place of the muscle fibers. (**c**) Normally developed intestine. Arrows point transversal muscles surrounding the intestine. (**d**) The intestine of the ds*LsalMS* treated animal. Numerous empty, swollen granules are visible. The intestinal wall is thinner than in the control animal. The transversal muscles are abnormally developed (arrows). Majority of the tissue surrounding the intestine is missing. (**e**) Normally formed brain. (**f**) The brain of the ds*LsalMS* treated animal. The brain is smaller and nervous tissue content is reduced. Arrows indicate numerous empty spaces. (**g**) Normally developed subcuticular tissue. The tegumental gland (Tg) is visible. (**h**) The subcuticular tissue of the ds*LsalMS* treated animal. The tissue layer is thinner, numerous cells and the tegumental gland are missing or abnormally formed. Abbreviations: brain (Br), muscle (Mu), intestine (I), tegumental gland (Tg). Scale bar = 50 μm (**a**,**b**); 25 μm (**c**,**f**).

Representative animals (preadult II female and adult males) were sectioned and stained with toluidine blue. All animals treated with ds*LsalMS* were smaller than controls and revealed abnormal morphology compared to the controls. The most striking abnormality in both sexes was significantly reduced content of various tissues. A severe reduction of muscle tissue was evident, fibres of longitudinal muscle stretching from cephalothorax to genital segment were discontinuous with visible ectopic tissue (Fig. 7a,b). The intestinal wall contained numerous vacuoles, and the connectivity between cells seemed affected (Fig. 7c,d). Not all cell types were identified in the intestinal wall of ds*LsalMS* treated animals, and circular muscles surrounding the intestine were thinner and lacked surrounding tissue (Fig. 7c,d). Numerous vacuoles were present in the brain. Moreover, the brain was smaller and empty spaces were visible in the inner part, where *Lsal*MS was localized (Fig. 7e,f). The cellular structures in the subcuticular tissue were strongly reduced, and separated with wide empty spaces. Numerous cells and the tegumental glands are missing or abnormally formed (Fig. 7g,h).

**Figure 8:**
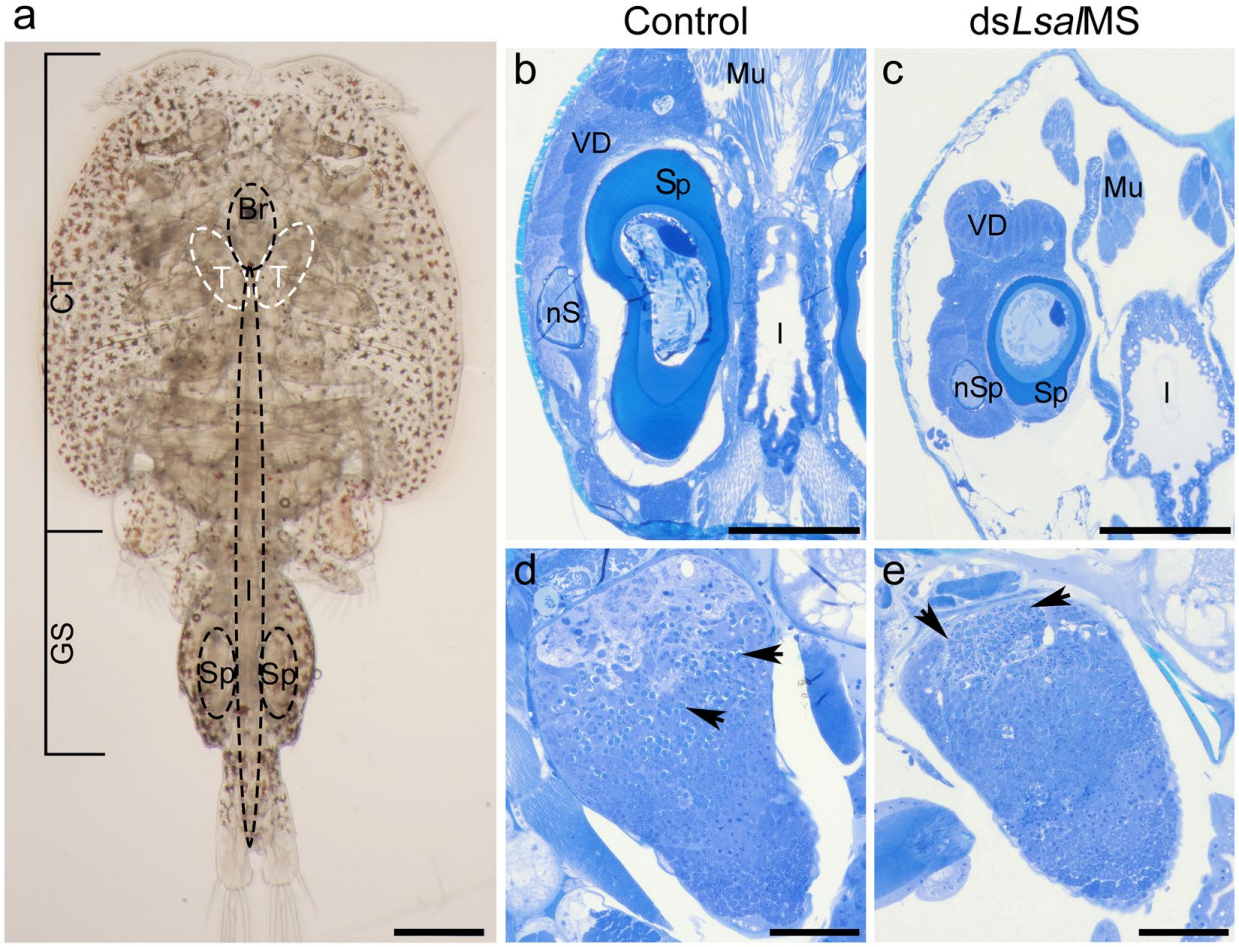
Histological sections of adult males. (**a**) Dorsal view of adult male. The brain and testes are located in the cephalothorax (CT), and spermatophores (Sp) in the genital segment on both sides of the intestine (I). Sections of control (**b**,**d**) and ds*LsalMS* treated male (**c**,**e**). Coronal sections. Images are taken in the corresponding locations in the control and the ds*LsalMS* treated animals. (**b**) Normally developed genital segment of adult male, with mature spermatophore (S) and spermatophore in formation process (nS). Genital segment is packed with various tissue types. (**c**) The genital segment of the ds*LsalMS* treated adult male. The majority of tissue is missing or abnormally developed. Striking are wide and empty spaces between organs. Mature spermatophore and spermatophore in formation process are present, whereas, they are reduced in size and misshaped. (**d**) Normally developed testicle. (**e**) The testicle of the ds*LsalMS* treated adult male is smaller. The lumen of the tubules seems thinner and tubules are more packed. In the anterior part of the testicle numerous “A” granules are visible (arrows). Abbreviations: spermatophore (Sp), newly forming spermatophore (nSp), muscle (Mu), intestine (I), vasa deferentia (VD). Scale bar = 500 μm (**a**), 50 μm (**b**,**c**); 10 μm (**d**,**e**).

In the transverse section of the male genital segment large, empty spaces were visible (Fig. 8b,c). The spermatophores at different stages of maturity were still present but they were abnormally shaped and smaller compared to the controls. While all typical layers of spermatophore wall were identified, they were much thinner than in the control animals (Fig. 8b,c). Males treated with ds*LsalMS* had smaller testes, with tubules tightly compressed. In the posterior zone of testes, spermatogonia were visible and the midzone contained primary spermatocytes. However, the anterior zone with spermatids contained a higher number of “A” granules than in control animals (Fig. 8d,e). The “A” granules are normally formed from degenerating spermatocytes^57^, indicating increased degeneration process in ds*LsalMS* treated males.

**Figure 9:**
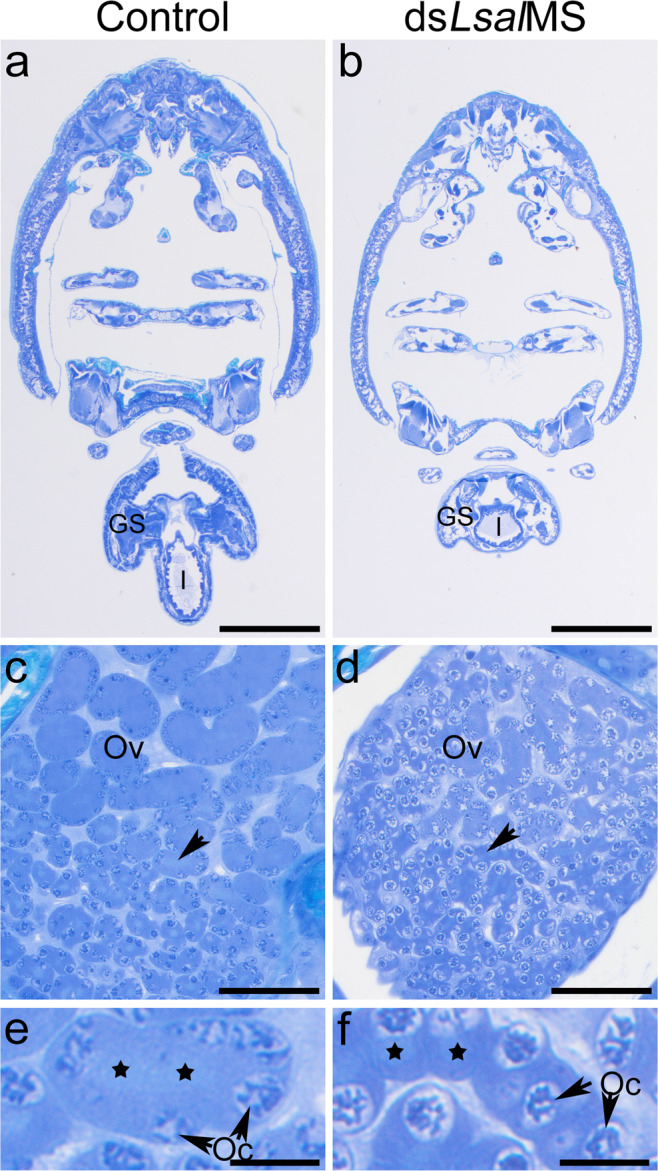
Histological sections of preadult II female. For description of anatomical structures in adult female lice see Fig. 4a. Control (**a**,**c**,**e**) and ds*LsalMS* treated preadult II female (**b**,**d**,**f**). Coronal sections, dorsal view, anterior at the top. Images are taken in the corresponding locations in the control and the ds*LsalMS* treated animals. (**a**) The whole body section of the normally developed preadult female II and (**b**) ds*LsalMS* treated. ds*LsalMS* treated preadult II females were smaller with lower density of tissue throughout the body. The genital segment is significantly reduced. (**c**) Normally developed ovary and (**d**) smaller ovary of the ds*LsalMS* treated preadult II female. The connective tissue normally located between tubules is reduced. Tubules do not expand in the anterior part of the ovary. Arrows indicate the lumen of the tubules. (**e**) The close up of the normally developed ovarian tubule pointed with arrow on (**c**), (**f**) the close up of the tubule of the ds*LsalMS* treated preadult II female pointed on (**d**). The lumen of the tubules (asterisks) is thinner and tubules are more compressed. Single oocytes pointed with arrowheads. Abbreviations: genital segment (GS), intestine (I), ovary (Ov). Scale bar = 100μm (**a**,**b**); 50 μm (**c**,**d**); 10 μm (**e**,**f**).

*LsalMS* knock-down preadult II females were generally frailer than corresponding controls, with reduced tissue content in the whole body (Fig. 9a,b). Moreover, the ovaries of ds*LsalMS* treaded females showed substantial changes. The ovaries were smaller and the ovarian tubules thinner and their lumen narrower compared to control animals (Fig. 9c–f). The chromosomes in the oocytes were clearly visible, but the nuclei seemed to lack nucleoplasm (Fig. 9e,f).

## Discussion

Myosuppressin peptide has previously been detected in many Arthropod species, but was reported to be missing in the salmon louse *Lepeophtheirus salmonis*^16^. In this study, we report for the first time, the identification of a myosuppressin peptide and characterisation of its deficiency in various developmental stages of the salmon louse. Compelling evidence shows that *Lsal*MS is essential for normal development, reproduction, and feeding of this economically important parasite. Moreover, to our knowledge this is the first report describing complex effects of myosuppressin deficiency on normal development throughout the life cycle in a single species.

The sequence analysis of *Lsal*MS revealed that this peptide belongs to the FMRFamide related peptide family (FaRPs) with the decapeptide sequence following the general pattern characteristic for myosuppressins identified in other Arthropod species. Protein sequence analysis revealed that the putative decapeptide of *L. salmonis*, pEDVDHVFLRFamide, is flanked by abnormal proteolytic cleavage sites of tribasic and dibasic amino acids, therefore, unusual processing into the active substance could be involved^58,59^. While the final mature sequence of *Lsal*MS needs to be experimentally validated in the future, the predicted one varies with only one amino acid on the N-terminal end of the peptide from leucomyosuppressin, LMS – QDVDHVFLRFamide^20,21^, and it is identical to one of the peptides from the beetle *Zophobas atratus*^14^.

Only one gene encoding MS has been identified in Arthropod species, and in the majority of these only one peptide product has been found^20,22–24,26–30^. However, despite of the single copy of the MS gene, in a few species additional copies of MS peptide products have been detected, with distinct tissue-specific distribution and function^14,53,60,61^. The exact mechanism of their production is unclear in most of the cases but post-transcriptional modifications and alternative processing sites could play a role^60^. In our study, only one gene encoding myosuppressin was found in salmon louse, with a single putative decapeptide released from the C-terminal end of the precursor. No alternative splicing or post-transcriptional modifications were detected, thus it seems that a single molecular form could account for all MS activity in *L. salmonis* tissues.

MS transcript level and tissue-specific distribution have been reported in a number of species, mainly in adult animals^34,62–65^, but to a lesser extent in larvae^34,51,62^. However, none of these studies have investigated the *MS* levels during the whole life cycle for any species. *LsalMS* expression was detected in all developmental stages. *LsalMS* transcripts can be detected just after hatching, in the nauplius I stage, with slightly higher expression in nauplii II stage, to reach the highest (13-fold higher than in nauplii I) expression in the copepodids (Fig. 3). After infection of the host, expression of *LsalMS* gene decreases gradually until adulthood, with adults expressing the lowest level of *LsalMS*.

The significant variation in the transcript level between copepodid and remaining stages suggests the increased requirement for *Lsal*MS activity in this planktonic and infectious stage. Copepodid is the transitional stage between the free-living and the parasitic phases in salmon louse ontogeny. The development of lice is arrested at this stage until the copepodid finds a suitable host i.e. a salmonid. The successful settlement on the host fish is a critical factor that can interrupt arrest and enable further development. It has been shown that myosuppressin is involved in the regulation of diapause in the cabbage army moth *Mamestra brassicae*^51^, and the concentration of myosuppressin in the hemolymph after pupal ecdysis was higher in diapause pupae than in nondiapause pupae. The myosuppressin suppresses activation of the prothoracic glands (PGs) stimulated by prothoracicotropic hormone leading to diapause^51^. It is therefore possible that *Lsal*MS could possess a similar function in copepodid stage of *L. salmonis*.

The analysis of the tissue specific peptide distribution suggests that *Lsal*MS plays a role in feeding/digestion and reproduction in *L. salmonis*. The presence of the *myosuppressin* transcripts in the tissue surrounding vital organs collected from the adult lice was also validated by quantitative RT-PCR. As expected, the highest transcript level was identified in the extracts from the brain, followed by mouth, intestine, and the reproductive organs: testes and ovaries. The observed *LsalMS* distribution is consistent with previously reported from other Arthropod species, where the MS peptide was detected in the central nervous system, peripheral neurons and endocrine glands^22,50,51,62,65^. Similarly to our results in *L. salmonis*, the MS-positive nerves were identified in close proximity of the crop muscle, in the crop nerve and in ramifying nerve fibres on the surface of the crop in *Drosophila suzukii*^64^. Furthermore, in *Rhodnius prolixus*, MS-positive nerves were detected in the endocrine-like cells in the midgut^50^. However, previous studies did not reveal MS-positive neurons or endocrine-like cells in the reproductive system. In *L. salmonis*, MS-positive cells were detected in the lumen of tubules of the ovaries. This suggests that in various species MS may play its role in distinct routes, acting as a neurotransmitter directly delivered to the target tissue and/or as a circulatory hormone, released from endocrine-like cells. Nevertheless, the localisation of the *Lsal*MS was consistent with previously described physiological function in regulating contractions of the various visceral muscles and support claims that *Lsal*MS, as myosuppressins in other species, is likely the neuromodulator in the nervous system and plays a role in regulating feeding and reproduction.

Very few data from RNA interference experiments assess effects of the myosuppressin deficiency in Arthropods. In females of *B. germanica*, RNAi mediated knockdown of LMS caused increase in the heartbeat frequencies^66^, whereas, nothing is known about the influence of the myosuppressin deficiency on the development and growth. Our RNAi studies indicate that myosuppressin directly or indirectly influences proper development, moulting and survival. The RNAi treated *L. salmonis* larvae, with absence of myosuppressin, were able to settle on the fish in the same rates as control animals, whereas, the development and further growth were affected significantly, with very few animals reaching the pre-adult stage. The survival was significantly reduced and only single animals, those where RNAi mediated knock-down was insufficient, survived to adulthood. Similar results were obtained when pre-adult lice were injected with ds*LsalMS*. Visual inspection of the intestine of ds*LsalMS* treated animals in all evaluated stages revealed significantly reduced or no gut content, indicating disturbance of the feeding process. In fact, close examination of the intestinal peristalsis in the recovered ds*LsalMS* treated preadult II females revealed significant increase in the contraction frequency. Moreover, lice had difficulties with moulting to the next stage, which could be connected to impaired coordination of muscle contractions. The observed phenotype was most severe in the female lice, which were unable to reach the adult stage, therefore, the exact effect of myosuppressin deficiency on reproduction of female lice could not be examined. However, very few male lice reach the adult stage and the complex malformations could be observed, mainly in the genital segment. Adult ds*LsalMS* treated males placed together with non-injected adult females were not able to complete mating. This effect clearly indicates that lack of *Lsal*MS, apart from other defects, significantly affects mating and reproduction in *L. salmonis* males. These results are consistent with previously reported function of myosuppressin in reproduction and feeding^45,63,64,67^.

The histological analysis of the adult males and preadult II females indicated developmental abnormalities in several tissues. The most striking feature was severe reduction of the muscle content, with a significant reduction of circular muscles in the intestine, as well as the visceral muscles associated with the vasa deferentia in the genital segment. Moreover, abnormalities in the skeletal muscles were apparent, with discontinuous fibres disturbed by the ectopic tissue. While the function of the myosuppressin on the activity of visceral muscles is well investigated, the influence of deficiency of myosuppressin on muscles development is poorly understood. Moreover, there is very scarce information available regarding myosuppressin function in the skeletal muscles, suggesting that it enhances the force of neurally induced contractions in skeletal muscle^31^. The results from the study in the cockroach *Periplaneta americana* indicates that the muscles of the legs receive innervation by FMRFamide-related peptide-containing neurons and that may be an additional method of modulating the mechanics of skeletal muscle contraction^68,69^. These findings indicate that myosuppressin has distinct effects on the different types of muscles, inhibiting contractions of the visceral muscles and enhancing the force of contractions in the skeletal muscles.

It was previously shown that muscle mass can dramatically decline in response to pathological signals or lack of normal neuronal signalling^70–72^, suggesting that the significant reduction in the content of the muscle tissue in myosuppressin deficient animals could be potentially linked with a lack of the proper neuronal stimulation. In contrary, *Lsal*MS deficiency affects intestinal morphology and function, likely leading to the reduced nutrient uptake, causing decreased tissue development and growth. Inability to acquire nutrients, malnutrition and starvation are well known causes of muscle atrophy in various species^73–75^, manifested as decrease in the body size and weight, cell death, intracellular vacuolation and increased intracellular spaces^73,74^. Due to various potential reasons of the observed phenotype in *Lsal*MS deficiency *L. salmonis*, the exact reasons of muscle mass decline need to be study further.

Data presented here suggests that the *Lsal*MS plays an important role in various physiological processes that are crucial for development, growth and behaviour in the salmon louse. Considering the importance this peptide has in function of vital organs in arthropods, it is not surprising that lack of *Lsal*MS causes major phenotype. The knock-down of *LsalMS* affects several processes that are critical for survival, like feeding and reproduction. The myosuppressin peptide could be a valuable target for the development of the new and effective anti-lice control method, that could effectively reduce feeding success and reproduction rate of lice and decrease the overall growth of sea lice populations in salmon cages, while targeting adult stages, and significantly decrease the number of lice reaching the adulthood, while targeting myosuppressin signalling in early developmental stages. However, further studies are required in order to unravel the complex roles played by *Lsal*MS in *L. salmonis* development, peptide processing and maturation pathways and interactions with specific receptors to find out how this peptide could be successfully targeted.

## Materials and Methods

### Ethics statement

The experiments were carried out on salmon louse *Lepeophtheirus salmonis*, whereas fish Atlantic salmon (*Salmo salar*) were used only as a host for the experimental animals. All procedures involving animals were performed according to The Norwegian Animal Welfare Legislation. The Animal Ethics Committee by The Norwegian Food Safety Authority approved all experiments.

### Salmon lice culture

A laboratory *Ls*Gulen strain of salmon lice (*Lepeophtheirus salmonis salmonis*) was maintained on farmed Atlantic salmon (*Salmo salar*)^76^. Salmon were hand fed on a commercial diet and reared in seawater with a salinity of 33.5‰ and a temperature of 12 °C. Eggs, nauplii and copepodids were kept in seawater from the same supply. Nauplii were obtained from hatching eggs and kept in single wells in a flow through incubators^76^.

### Sequence analysis

Candidate *L. salmonis* myosuppressin peptide gene was identified by blasting (BLASTP) the known propeptide sequence of *Drosophila melanogaster* (AAF56283.1) against collection of predicted protein sequences from the salmon louse (sealouse.imr.no). Only one high confidence hit was identified (e-value = 7e^−08^). Full-length cDNA sequence was obtained by rapid amplification of cDNA ends (RACE) using SMARTer®RACE cDNA Amplification Kit (Clontech) according to the manufacturer’s protocol. RACE primers (Supp. Table 1) were designed based on the sequence retrieved from salmon louse genome (sealouse.imr.no), giving 279 bp overlapping fragments at the ends of 5′ and 3′ RACE products (Fig. 1a). Obtained fragments were cloned using the TOPO TA Cloning® Kit for sequencing (Invitrogen) and sequenced with M13 forward and reverse primers and sequence specific primers used for RACE (Supp. Table 1). Prior to sequencing the PCR products were purified by ExoSAP-it (Affymetrix). Obtained sequences were assembled using MacVector with Assembler vs. 12.7.5 (MacVector, Inc, Software for Scientists, North Carolina, USA). Final sequence of *Lsal*MS transcript, encoding prepropeptide was submitted to the GenBank. *Lsal*MS gene organisation was analysed by aligning obtained cDNA sequence of *Lsal*MS with salmon louse genome sequence (sealouse.imr.no). A precursor peptide sequence was predicted by identification of a signal peptide cleavage site using SignalP 4.1 software (http://www.cbs.dtu.dk/services/SignalP/)^77^. The mature peptide sequence was identified by multiple sequence alignment of the amino acid sequences (accession numbers are listed in Table 1) performed with ClustalOmega^78^.

### Phylogeny

The full length of *Lsal*MS predicted precursor peptide sequence was compared to 20 precursors of myosuppressins belonging to other species. Propeptide sequences used in the present study were downloaded from NCBI (http://www.ncbi.nlm.nih.gov/) and their accession numbers are listed in Table 1. Putative domains of *Lsal*MS were predicted and examined by Pfam (http://pfam.xfam.org/). Multiple alignment of the propeptides amino acid sequences was performed with ClustalOmega^78^, and converted to nexus format by Mesquite v. 3.2^79^. LG + G + I model of substitution was adopted as the optimal evolutionary model, which was predicted by ProtTest 3.4^80^. Phylogenetic analysis was conducted using MrBayes v.3.2^81^. Markov Chain Monte Carlo (MCMC) algorithm was used with four chains running for 1,000,000 generations and sampled every 1000 generations. Output data were analysed with Tracer v1.5^82^, and tree was obtained using FigTree v1.4.3 (http://tree.bio.ed.ac.uk/software/figtree/).

### Collection of animals and tissues for ontogenetic analysis

The following numbers and groups of animals were collected in quadruple samples. Stages: nauplius I, nauplius II and copepodid (free-living) – approximately 200 larvae per sample, chalimus I – 30 animals, chalimus II – 20 animals, preadult I, II and adult stages – single animals. Specific tissue and organs were dissected from 50–100 animals per sample from both sexes (brain, mouth, intestine) or only from one corresponding sex (ovaries, testes, genital segments).

### Immunohistochemistry

For detection of *Lsal*MS peptide, the following immunohistochemistry protocol was used. Briefly, slides with paraffin sections were incubated at 65 °C for 30 min and afterwards cleared in Histo-Clear II (National Diagnostics) three times for 10 min, following by rehydration in decreasing ethanol series (2X 100%, 96%, 80%, 50%) for 3 min each and then put into distilled water. After a heat-induced antigen retrieval (HIER) (30 min in a 95 °C sodium citrate buffer (10 mM Sodium Citrate, 0.05% Tween 20, pH = 6.0))^83^, slides were washed two times for 10 min in PBST (Phosphate buffered saline, Sigma Aldrich, with 1% Triton–X) on the shaker. 2% NGS (Normal Goat Serum, Jackson ImmunoResearch Lab) and 2% BSA (Bovine serum Albumin; Sigma-Aldrich) in PBST was used to block unspecific antiserum binding for 2 h at room temperatures. Primary antiserum was diluted 1:500 in PBST with 2% BSA/2% NGS and left on the sections overnight at 4 °C, in the dark. Polyclonal rabbit anti-MS, the gift form Dr. A. Urbański, were previously used in^62,65^. Incubating tissues without peptide antiserum was performed as a control. After five washes in PBST (10 min each) and one overnight wash at 4 °C, the secondary antibody (1:100, diluted in TBST; Anti-Rabbit IgG (whole molecule)–Alkaline Phosphatase antibody produced in goat, Sigma Aldrich) was applied for 1 h at room temperature. After five 10 minutes washes in PBST on the shaker, the slides were washed three times 10 minutes with processing buffer (100 mM Tris-NaCl, 50 mM MgCl2, pH 9.5) and then incubated in NBT-BCIP solution (Sigman Aldrich). As soon as staining became visible, the reaction was stopped by incubation in distilled water. Sections were mounted using ImmunoHistomount (Sigma Aldrich) and photographed using Zeiss Axicam 105 color (Zeiss) camera mounted with to Zeiss Axio Scope.A1 (Zeiss) microscope.

### Production of dsRNA

*LsalMS* gene sequence was too short to test two independent dsRNA fragments. Therefore, only one dsRNA fragment was tested in this study, complementary to the gene sequence that shows lowest similarity to other salmon louse sequences. To test it, selected sequence was blasted (BLASTN) against sea lice genomic sequence and collection of predicted transcripts (sealouse.imr.no). Templates for dsRNA were generated in two steps. First, PCR products were obtained from copepodids cDNA for *LsalMS* and plasmid containing cod *CPY185* gene for control fragment with the specific pairs of primers (Supp. Table 1). Later, the final templates were generated with the same primers but with T7 extensions added (Supp. Table 1). dsRNA synthesis was performed with T7 RNA Polymerase from MEGAscript® RNAi Kit (Ambion Inc.) according to manufacturer instructions. Obtained dsRNA fragments were 279 bp long for *LsalMS* and 800 bp for *CYP185*. Area of *LsalMS* targeted by dsRNA is indicated on Fig. 1a. Final concentration of dsRNA was measured with spectrometry (NanoDrop Technologies Inc.).

### RNA interference

RNAi on nauplius I larvae was performed as described earlier^84^. The experiment was executed using groups consisting of approximately 2000 nauplius I larvae. For soaking, these were divided in subsamples of 100 animals, each incubated in 200 μl of seawater in the lid of a 1.5 ml microcentrifuge tube. dsRNA was added to the final concentration of 20 ng/μl. The existence of any unspecific effects of soaking nauplii in dsRNA on gene expression was assessed by soaking animals in the control dsRNA, containing a fragment with no significant sequence similarity to the salmon louse genomic sequence (a cod trypsin gene, *CPY185*)^85^. Animals were incubated overnight. When moulting was finished, all animals within each treatment group were pooled and transferred to flow through incubators, where they were kept until sampling on day 7 post hatching. Before sampling, lice were inspected under a binocular microscope (Olympus SZX12, 0.5× Olympus objective). Photographs of copepodids were taken using a Zeiss Axicam 105 color (Zeiss) camera mounted to Zeiss Axio Scope.A1 (Zeiss) microscope. For fish infection, 12 groups of 100 copepodids from each treatment group were separated. For total RNA extraction copepodids were divided into five parallels with around 100 animals per sample and preserved in RNAlater (Invitrogen). For histological analysis, copepodids were collected in Karnovsky fixative.

RNAi on preadult I females and males was performed as previously described^85^. dsRNA fragments were diluted to a concentration of 600 ng/μl and bromophenol blue was added to visualise dsRNA solution during injection. On the day of injection, preadults I of both sexes were collected from the fish and injected with 0,5 μl of dsRNA solution in cephalothorax, with care not to disturb the intestine. After injection, the animals were incubated in seawater for around 4 h and placed back on fish. For each fragment, in total eight fish were used, each carrying 20 injected preadult I females and 20 injected preadult I males. A cod trypsin dsRNA *CPY185* fragment was used as a control. The experiment was terminated after 3 weeks, when all ds*LsalMS* treated female were lost from the fish. To evaluate mating potential of adult males, a total of 30 pre-adult I male lice were injected with ds*LsalMS*, and placed together with non-injected females (10 males and 10 females per fish, fish n = 3). The same experimental set up was repeated for males injected with control dsRNA. The experiment was terminated after 6 weeks, when all control female lice had developed at least their first pair of egg strings. At termination, all recovered lice were subjected to a gross morphological inspection before lice were sampled for RNA extraction in RNAlater or histological analysis in Karnovsky fixative. Photographs were taken with Canon EOS 600D camera mounted with an adaptor (LMscope) to an Olympus SZX9 dissecting microscope.

For evaluation of the intestine contraction rate, preadult II females were placed on petri dish in the drop of seawater and intestinal movement was recorder for 1 minute at a time, using Canon EOS 600D camera mounted with an adaptor (LMscope) to an Olympus SZX9 dissecting microscope. Each louse was recorded twice. Number of contractions was counted on each movie. (Samples of the movies in Supp. Video 1).

### Infection of host fish

Atlantic salmon (*Salmo salar*) were placed in single fish tanks (70 litres) with constant flow of seawater^76^. Seawater temperature was maintained at 12 °C during the whole experiment. 12 fish were infected with copepodids treated with ds*LsalMS* and 12 with control copepodids treated with ds*CPY185*. During infection period, water level was decreased to around 15 cm, to enhance contact between fish and copepodids. Fish were washed by 100 copepodids (7–9 days post hatching) and left without water flow for 10 minutes, with water manually mixed after 5 minutes. After that time, the water flow was restarted. Lice were collected from four fish from each group after 8, 12 and 18 days post infection, when control lice were in chalimus I, chalimus II and preadult stages, respectively. At termination, all recovered lice were subjected to a gross morphological inspection, counted and staged. Photographs were taken with Canon EOS 600D camera mounted with an adaptor (LMscope) to an Olympus SZX9 dissecting microscope. Lice were sampled for RNA extraction in RNAlater or histological analysis in Karnovsky fixative.

### RNA extraction and cDNA synthesis

Total RNA was extracted as described earlier^86^. Total RNA was diluted in DEPC-treated water (Invitrogen) and the final concentration was evaluated with spectrometry (NanoDrop Technologies Inc.). From each sample 2000 ng of total RNA was DNAse treated in 10 μl reaction (TURBO^TM^ DNase, Ambion). 3 μl of each DNAse treated total RNA was used in cDNA synthesis with oligo(dT) and random primers, using program recommended by the manufacturer: primers annealing: 5 min −25 °C, synthesis: 45 min −42 °C, termination: 5 min −95 °C (AffinityScript qPCR cDNA Synthesis Kit, Stratagene). cDNA was diluted four times before use in qRT-PCR or storage at −20 °C. 2 μl of final dilution was used in each PCR reaction.

For evaluation of *LsalMS* transcript level in chalimus I and chalimus II Direct zol RNA Microprep Plus (ZYMO Research) kit was used, according to the manufacturer instructions. TotalRNA from single preadult I lice was extracted using Tri Reagent (Sigman Aldrich) as described before^86^.

### Quantitative RT-PCR (qRT-PCR)

qRT-PCR was performed and analysed using the *elongation factor 1 alpha* (*EF1α*)^87^ and filamin A^88^ as reference genes. Both genes are considered to be good references, with stable expression rate in all developmental stages and under RNAi treatment conditions^86,88^. All primers and probes are listed in Supp. Table 1. All qRT-PCRs were carried out in technical duplicates. The assays were performed simultaneously for the reference and test genes using the same cDNA and master mix (PowerUp® SYPR®Green Master Mix; Applied Biosystems). Thermal cycling and quantification were done on the QuantiStudio3 qPCR Machine (Applied Biosystems) in 10 μl reactions under standard conditions (initiation: 2 min −50 °C, holding: 2 min −95 °C, 40 cycles of 15 s −95 °C, 1 min −60 °C, followed by the Melt Curve Stage). Amplification efficiencies of target and reference genes were evaluated (*ef1α* – 93,8%, filamin A – 105,7%, *LsalMS* – 104,6%). Relative expression was calculated using 2^−ΔΔCT^ formula, with amplification efficiency correction for each gene (Pfaffl Method), utilizing the controls of each experiment as a standard. Independent-Samples T-tests were used to determine if control and test groups were differently expressed. A p-value of 0.05 was chosen as threshold.

### Histology

Animals were fixed in Karnovsky fixative, washed twice in PBS, dehydrated in a graded ethanol series, pre-infiltrated with Technovit7100/ethanol (50/50) for 4 hours (Technovit 7100, Heraeus Kulzer Technique) and infiltrated with Technovit7100/hardener overnight before embedding in plastic. Two μm thick sections were produced using a microtome (Leica RM 2165) and stained with toluidine blue (1% toluidine blue in 2% borax) for 30 s. The stained sections were mounted using DPX New Mounting Medium (Merck). Photographs were taken using a Zeiss Axicam 105 color (Zeiss) camera mounted to Zeiss Axio Scope.A1 (Zeiss) microscope.

### Statistical analysis

Statistical evaluation was performed by Independent-Samples T-Test.

## Supplementary information

Primer sequences used in this study

Contractions of the intestinal wall in female lice after RNAi experiment
