# Author Correction: RNAi mediated myosuppressin deficiency affects muscle development and survival in the salmon louse (*Lepeophtheirus salmonis*)

**DOI:** 10.1038/s41598-020-69891-2

**Published:** 2020-09-23

**Authors:** Anna Z. Komisarczuk, Heidi Kongshaug, Ming Li, Frank Nilsen

**Affiliations:** 1 Sea Lice Research Centre, Department of Biological Sciences, University of Bergen, Thormøhlensgate 53 A/B, 5008 Bergen, Norway; 2 Key Laboratory of Aquaculture Disease Control, Ministry of Agriculture, and State Key Laboratory of Freshwater Ecology and Biotechnology, Institute of Hydrobiology, Chinese Academy of Sciences, Wuhan, 430072 China

Correction to: *Scientific Reports* https://doi.org/10.1038/s41598-019-43515-w, published online 06 May 2019

In the original version of this Article, Supplementary Video 1 was published in an incorrect format.

In addition, the email address for Anna Z. Komisarczuk quoted in the original version of this Article has been changed. Correspondence and requests for materials should be addressed to a.z.komisarczuk@ibv.uio.no.

These errors have now been corrected in the HTML and PDF versions of the Article, and in the Supplementary Information that now accompanies the Article.

