# Supplementary material for: RNAi mediated myosuppressin deficiency affects muscle development and survival in the salmon louse (Lepeophtheirus salmonis)
Source: Sci Rep. 2019 May 6;9:6944. doi: 10.1038/s41598-019-43515-w (PMC6502818; doi:10.1038/s41598-019-43515-w)
Supplement: Supplementary file 1 — Primer sequences used in this study [file 41598_2019_43515_MOESM1_ESM.docx]

RNAi mediated myosuppressin deficiency affects muscle development and survival in the salmon louse (*Lepeophtheirus salmonis*)

Anna Z Komisarczuk, Heidi Kongshaug, Ming Li, Frank Nilsen

**Supplementary Table 1**

| Primer name | Sequence (5’ 🡺 3’) | Method |
| --- | --- | --- |
| M13_f | GTAAAACGACGGCCAG | TOPO Cloning |
| M13_r | CAGGAAACAGCTATGAC | TOPO Cloning |
| *Lsal*EF1a_f | GGTCGACAGACGTACTGGTAAATCC | RTq-PCR |
| *Lsal*EF1a_r | TGCGGCCTTGGTGGTGGTTC |  |
| *Lsal*FilA_f | CGGAACGTTTTCCCTAAACA | RTq-PCR |
| *Lsal*FilA_r | CGGGTCCATACACTCGAACT |  |
| *Lsal*MS_qPCR_f | CAACCCTTTTGAGAGGCAAA | RTq-PCR |
| *Lsal*MS_qPCR_r | CAACAAGGAGTGGTGTTGAGC |  |
| *Lsal*MS_f | GTTCCCCAAGTCCTCTTTCC | dsRNA, RACE |
| *Lsal*MS_r | GCTTGGTGGTTGTCGTAGGT |  |
| *Lsal*MS_T7_f | TAATACGACTCACTATAGGGagaGTTCCCCAAGTCCTCTTTCC | dsRNA |
| *Lsal*MS_T7_r | TAATACGACTCACTATAGGGagaGCTTGGTGGTTGTCGTAGGT |  |
| Cod_CYP185_f | ATAGGGCGAATTGGGTACCG | dsRNA |
| Cod_CYP185_r | AAAGGGAACAAAAGCTGGAGC |  |
| Cod_CYP185_T7_f | TAATACGACTCACTATAGGGagaATAGGGCGAATTGGGTACCG | dsRNA |
| Cod_CYP185_T7_r | TAATACGACTCACTATAGGGagaAAAGGGAACAAAAGCTGGAGC |  |

**Supp. Table 1. Primer sequences used in this study.** All primers were provided by Sigma-Aldrich, St Louis, MO, USA. T7 extension (TAATACGACTCACTATAGGGAGA, underlined) was added to primers specific for *LsalMS* and Cod specific *CYP185* primers in order to generate dsRNA.
