# Supplementary material for: RNAi mediated myosuppressin deficiency affects muscle development and survival in the salmon louse (Lepeophtheirus salmonis)
Source: Sci Rep. 2019 May 6;9:6944. doi: 10.1038/s41598-019-43515-w (PMC6502818; doi:10.1038/s41598-019-43515-w)
Supplement: Supplementary file 2 — Contractions of the intestinal wall in female lice after RNAi experiment [file 41598_2019_43515_MOESM2_ESM.pptx]

## Slide 1
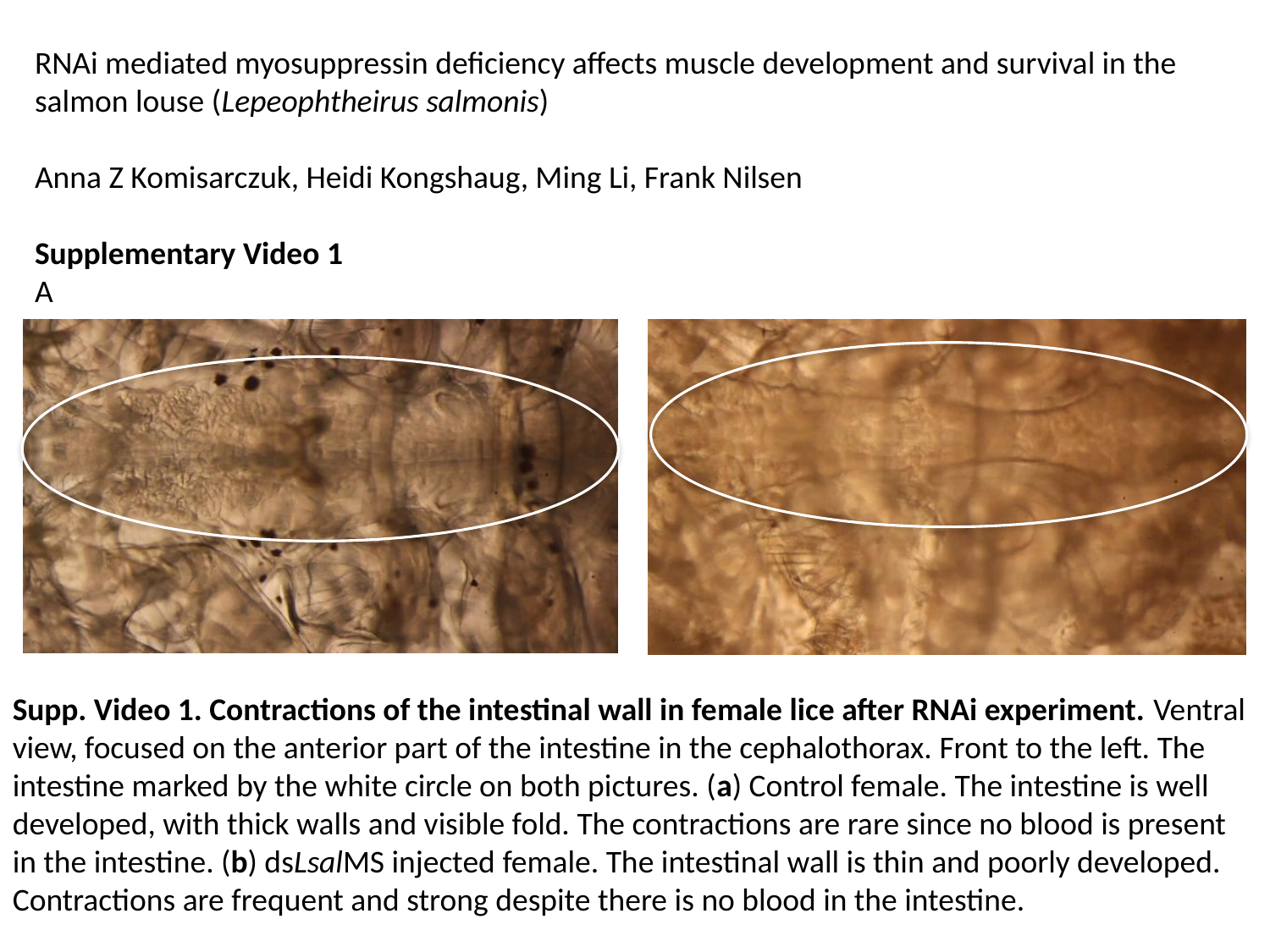

RNAi mediated myosuppressin deficiency affects muscle development and survival in the salmon louse (Lepeophtheirus salmonis)
Anna Z Komisarczuk, Heidi Kongshaug, Ming Li, Frank Nilsen
Supplementary Video 1
A									 B
Supp. Video 1. Contractions of the intestinal wall in female lice after RNAi experiment. Ventral view, focused on the anterior part of the intestine in the cephalothorax. Front to the left. The intestine marked by the white circle on both pictures. (a) Control female. The intestine is well developed, with thick walls and visible fold. The contractions are rare since no blood is present in the intestine. (b) dsLsalMS injected female. The intestinal wall is thin and poorly developed. Contractions are frequent and strong despite there is no blood in the intestine.
